# Candida albicans Mastitis in Breastfeeding Woman: An Under Recognized Diagnosis

**DOI:** 10.7759/cureus.12026

**Published:** 2020-12-11

**Authors:** Yassine Merad, Hichem Derrar, Malika Belkacemi, Amine Drici, Zoubir Belmokhtar

**Affiliations:** 1 Parasitology-Mycology, "Hassani Abdelkader" University Hospital, Central Laboratory, Sidi Bel Abbès, DZA; 2 Pulmonary and Lung Diseases, "Hassani Abdelakder" University Hospital, Sidi Bel Abbès, DZA; 3 Hemobiology and Blood Transfusion, "Hassani Abdelakder" University Hospital, Sidi Bel Abbès, DZA; 4 Biology, Universty Djilali Liabes, Sidi Bel Abbès, DZA; 5 Biology, University Djilali Liabes, Sidi Bel Abbès, DZA

**Keywords:** candida mastitis, breastfeeding woman, areola hyperkeratosis, nipple discharge, candida albicans, nipple pain, breast mycosis, fungal diseases, clinical dermatology

## Abstract

Breastfeeding has been demonstrated to have many benefits for both mother and child. The diagnosis and management of Candida in the breastfeeding dyad are difficult because diagnosis is most often based on subjective signs and symptoms. Few studies have attempted to confirm the diagnosis with biological tests. A 26-year-old breastfeeding mother presented with stabbing breast pain, accompanied by erythematous, hyperkeratotic areola, and nipples. Histopathological results and direct mycological examination, followed by culture and auxanogram, revealed Candida albicans as an etiological agent of mastitis; the case was managed successfully by antifungal medication. Candida mastitis in lactating women is an under-recognized and under-treated cause of breast pain, hyperkeratosis over areola, and discharge of nipple should be assessed by biological tests. The hope is that with an increased awareness of this infection, more mothers will be appropriately treated and will thus be more likely to continue breastfeeding.

## Introduction

The nipple and areola skin is highly specialized and differs structurally from that of the neighboring chest wall. The openings of lactiferous ducts, sebaceous, and apocrine glands are the features of the nipple skin. The areola is almost glabrous with a few villous follicles, a few apocrine glands, clusters of large sebaceous glands, and the so-called tubercles of Montgomery [1].

Candida mastitis condition is called thrush; it is a fungal infection that forms on the nipples and breast. In the literature, there are few data about breast candidiasis; some authors claimed that mammary candidiasis is a medical condition without scientific evidence [2, 3]. Moreover, it is widely believed that free fatty acids on the skin surface hinder the growth of pathogenic fungi and sebum, or at least the products of its hydrolysis exert a fungistatic effect [4]. Maternal causes of mastitis are nipple problems (blocked milk ducts, damaged nipples), feeding problems (missed feeds, suboptimal positioning and attachment, an oversupply of milk, insufficient milk removal and engorgement), maternal health (fungal infection elsewhere, breast trauma, recent antibiotic use), and maternal food habits (consumption of dairy products and sweetened foods). Other external risk factors are pacifier and teat use [5, 6].

Achy breasts or shooting pains deep in the breast can occur during or after feedings. The most common signs are pink, flaky, shiny, itchy, cracked, or blistered nipples. Areola hyperkeratosis and nipple discharge are clinical features of Candida mastitis, mycosis fungoides, and nevoid hyperkeratosis [3, 7, 8]. Several different Candida species may be found in infants' oral cavities and as part of breast skin flora [9].

In the present case, histopathology and mycological tests were performed to prove the fungal origin and rule out neoplastic causes.

## Case presentation

**Figure 1:**
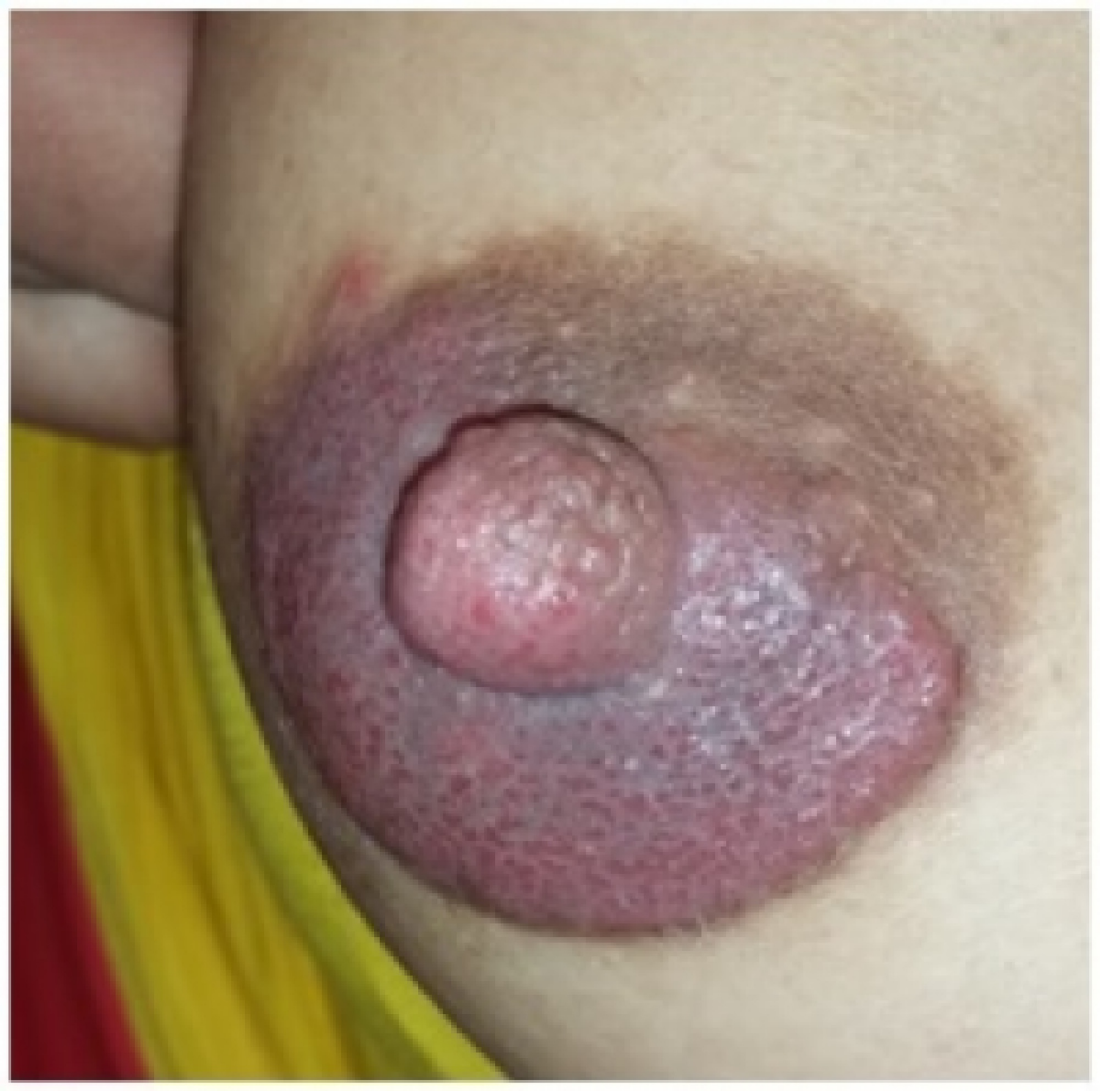
Patient’s breast manifestations (areola hyperkeratosis and discharge)

We report a 26-year-old breastfeeding mother admitted to our dermatology department for therapeutic management of breast disease. The patient presented with stabbing breast pain, accompanied by erythematous hyperkeratosis of the areola and nipples (Figure 1). She complained of itching over the right breast, gradually spreading for the last 20 days, and intermittent painful breastfeeding. There has no history of any topical or systemic medication and no family history of breast or ovarian cancer. The patient had three previous pregnancies and three deliveries; she reported that her child was six months old and that she continued to breastfeed him, and no artificial nipples were used. She reported that her infant son had oral thrush; the treatment was initiated about four weeks before her admission in our institution; no white spots on the inside of the baby’s cheeks, tongue, or gums were found at physical examination. It was found that the patient had a history of recurrent vaginal yeast infections. Her current condition was diagnosed as a yeast infection and was treated with miconazole vaginal cream.

**Figure 2:**
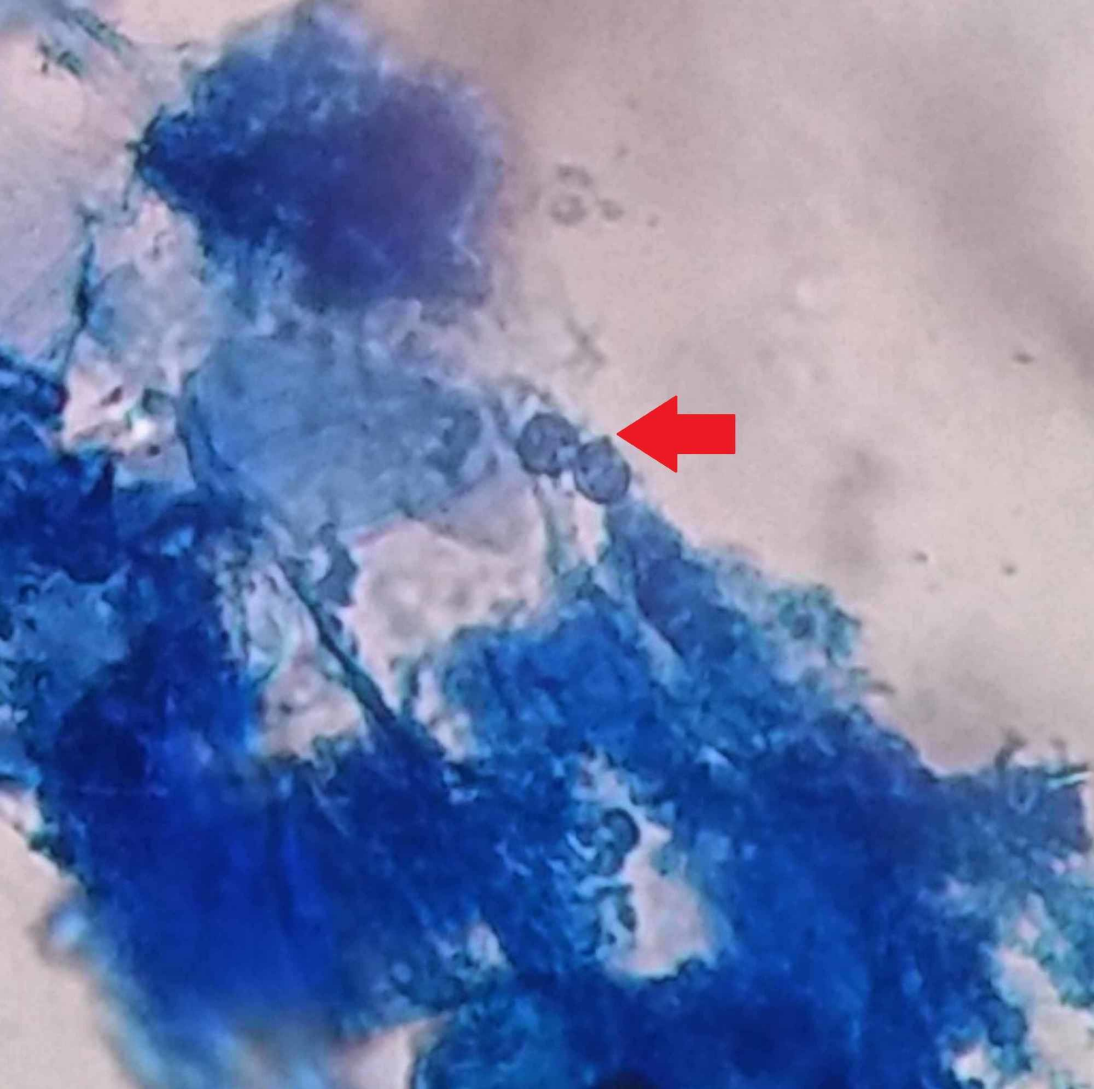
Lactophenol cotton blue wet mount showing budding yeast

Histopathology didn’t reveal any neoplastic disease and was in accordance with mycological examination of breast discharge and scraping of the areola. The direct microscopic examinations of obtained specimens were carried out to detect fungal elements by using potassium hydroxide (KOH) and lactophenol cotton blue wet mount (Figure 2). All specimens were inoculated onto duplicate tubes Sabouraud’s dextrose agar (SDA) and incubated at 25°C. Culture bottles were examined for the presence of growth after 48 hours.

**Figure 3:**
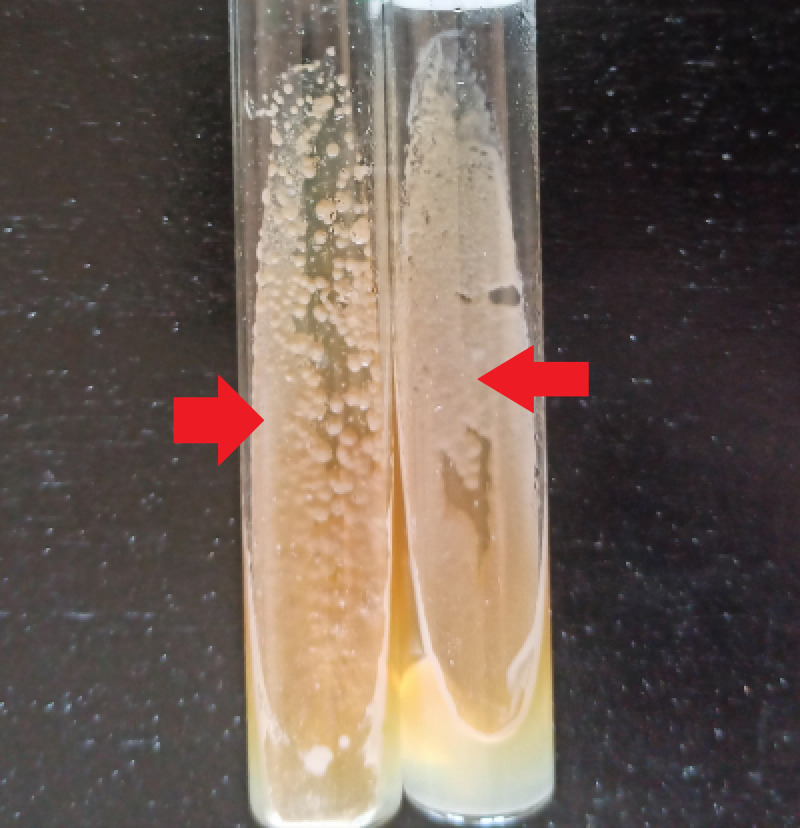
Creamy white culture of Candida albicans on SDA medium after 48 hours SDA - Sabouraud’s dextrose agar

On day 3, the results in Sabouraud’s culture (Figure 3) and biochemical test (Auxacolor©) were compatible with Candida albicans*. *On day 7, after laboratory tests (histology and mycology), the treatment was initiated - she was prescribed clotrimazole 1% topical cream and instructed to apply it after every breastfeeding session. Oral fluconazole was added for seven days. The patient showed responsiveness to treatment, and on completion of the therapy (day 15), she was asymptomatic.

## Discussion

Breastfeeding has many benefits for both mother and child [9]. In the literature, there are few data about Candida mastitis; among breastfeeding women in the US, 18% had nipple pain and Candida mastitis [10]. Pain is the second most common cause of breastfeeding discontinuation; this symptom usually occurs in the first six months after childbirth [9].

Many maternal risk factors of developing mastitis have been identified, including the consumption of dairy products and sweetened foods, which is frequent in our population. Moreover, the use of an antibiotic can lead to superficial mycosis and recurrent yeast infection (e.g., vaginal yeast infections) [5]. Our patient had a history of vaginal candidiasis treated by antifungals; the association of Candida mastitis with recurrent vaginal yeast infection was also previously described [9]. Other etiologic causes of Candida mastitis are trauma due to the infant’s mouth's incorrect attachment, the mouth's anatomy, or dysfunctional suck [6]. Moreover, artificial nipples are also contributing to breast colonization by Candida species [11]. Our patient did not use either a pacifier or a bottle. 

In practice, more than one cause of nipple or breast pain is commonly present [12]. This case revealed the presence of Candida albicans in breastfeeding woman, but persistent and strictly localized hyperkeratotic lesions of the nipple, areola, or both with unknown pathogenesis are signs of nevoid hyperkeratosis of the nipple and areola or mycosis fungoides; clinical features include verrucous thickening and brownish discoloration of the nipple [8]. Nipple thrush is usually diagnosed in postpartum when the nipple/areola is slightly pink, sensitive to touch, and the pain described is out of proportion to the damage seen on clinical examination [3, 7, 13].

Suitable biological tests do not document the most confirmed cases of mammary mycosis. Candida was found on the breasts of 34.55% of lactating women and 17.65% of non-lactating women; Candida albicans is at least identified by cultural data [11].

A publication results highlight the widespread colonization of Candida species both in infant’s oral cavities and on lactating mother's breasts, leading to plugged duct and mastitis [11]. Several things can cause plugging as severe engorgement or regular feeding from only one breast, and milk supply will often decrease. Resulting pain inhibits the let-down reflex, and babies with yeasts (in mouth) often do not nurse as efficiently as they do when their mouths are not sore. Few studies have attempted to confirm Candida mastitis diagnosis with biological tests; these exams are important to rule out nevoid hyperkeratosis of the nipple and areola or mycosis fungoides [7, 14, 15].

As proved by the high rates of Candida colonization in infants who use artificial nipples, eliminating the yeast on pacifiers and bottles is an important step of treatment [9]. A collaboration of lactation specialists and dermatologists showed that nystatin cream might be less effective than clotrimazole, ketoconazole, or miconazole [16]. As documented, patients with Candida mastitis were prescribed topical nystatin and oral fluconazole 150 mg for seven days; if the pain persists, oral nystatin (Mycostatin®) or fluconazole are recommended for at least two weeks for the breastfeeding woman. Nevertheless, it is often difficult to eradicate Candida mastitis if risk factors are not eliminated [9]. Breastfeeding has benefits for both mother and child. Thus, histopathology and mycological tests are required to prove fungal mastitis and to provide adequate treatment. 

## Conclusions

The diagnosis and management of Candida in the breastfeeding dyad are difficult because diagnosis is most often based on subjective signs and symptoms. Persistent and localized hyperkeratotic lesions of the nipple are signs of nevoid hyperkeratosis of the nipple and areola, mycosis fungoides, or Candida mastitis. Histopathology results and direct mycological examination, followed by culture, can reveal Candida albicans as the etiological agent of mastitis. With an increased awareness of this infection, more mothers will be appropriately treated and will thus be more likely to continue breastfeeding.
